# Acrophialophora jodhpurensis: an endophytic plant growth promoting fungus with biocontrol effect against *Alternaria alternata*

**DOI:** 10.3389/fpls.2022.984583

**Published:** 2022-09-23

**Authors:** Zoha Daroodi, Parissa Taheri, Saeed Tarighi

**Affiliations:** Department of Plant Protection, Faculty of Agriculture, Ferdowsi University of Mashhad, Mashhad, Iran

**Keywords:** beneficial endophytic fungus, early blight disease, plant growth parameters, *Solanum lycopersicum*, seed coating

## Abstract

In this study, efficiency of the endophytic fungal isolate Msh5 was evaluated on promoting tomato plant growth and controlling *Alternaria alternata*, the causal agent of early blight in tomatoes. Morphological and molecular (ITS and tub2 sequences) analyses revealed that the fungal isolate, Msh5, was *Acrophialophora jodhpurensis* (*Chaetomium jodhpurense* Lodha). This beneficial fungus was capable of producing indole-3-acetic acid (IAA), urease, siderophore, extracellular enzymes, and solubilized phosphate. Under laboratory conditions, the Msh5 isolate of *A. jodhpurensis* inhibited *A. alternata* growth in dual culture, volatile and non-volatile metabolites assays. The supernatant of this endophytic fungus was capable of reducing spore germination and altering the hyphal structure of *A. alternata* and the spores produced germ tubes showed vacuolization and abnormal structure compared to the control. Also, the effect of *A. jodhpurensis* on plant growth parameters (such as shoot and root weight and length) and suppressing *A. alternata* was investigated *in vivo* via seed inoculation with spores of *A. jodhpurensis* using 1% sugar, 0.5% carboxymethyl cellulose (CMC) or 0.5% molasses solution as stickers. Colonization of tomato roots by the endophytic fungus resulted in significant increasing plant growth parameters and reduction in the progress of the diseases caused by *A. alternata* compared to the controls. Among the different coating materials used as stickers, sugar was found to be the most effective for enhancing plant growth parameters and decreasing the disease progress. Therefore, *A. jodhpurensis* isolate Msh5 can be suggested as a potential biofertilizer and biocontrol agent for protecting tomato plants against *A. alternata*.

## Introduction

Tomato (*Solanum lycopersicum* L.) is one of the most important popular vegetables worldwide. Various species of *Alternaria* are causal agents of early blight disease, which is one of the major biotic stresses and reduces tomato production every year (Kumar et al., 2008). Yield losses due to tomato infection by this fungus are reported to be 30–79% (Dube et al., 2014) in different tomato growing regions.

The Alternaria genus, which was first introduced by Nees (1816), belongs to the kingdom Mycota, phylum Ascomycota, subphylum Pezizomycotina, class Dothideomycetes, order Pleosporales, family Pleosporaceae and is a ubiquitous necrotrophic fungus that includes saprobic, endophytic and pathogenic species (Saharan et al., 2016). The teleomorph of some Alternaria species is the genus *Lewia*. Fungi belonging to *Alternaria* spp. can grow on several substrates including all aerial parts of various plant species, agricultural products, soil, and air. Many species of Alternaria have been reported as the causal agents of tomato early blight disease, among them *A. alternata* is very important due to its destructive damage on the host plants (Ramezani et al., 2019).

The early blight is capable of causing damage at all growth stages and the disease symptoms occur on the leaves, fruits, and stems as brown and finally necrotic spots. Generally, the disease symptoms occur on the oldest leaves and cause severe destruction of the aerial part together with reduction in the size and number of tomato fruits (Fritz et al., 2006; Dharmendra et al., 2014). The fungal genus *Alternaria* produces large, multicellular, dark-colored conidia, in single or branched chains on short conidiophores (Thomma, 2003; Ramezani et al., 2019), which are important in infecting host plant tissues and disease distribution. *Alternaria* species are capable of residing in seeds and soil and the air-borne spores can be distributed over long distances, which make the disease control very hard.

To control plant diseases caused by *Alternaria* spp. various strategies can be used in agricultural systems. One of the disease management strategies is chemical control, which can prevent infection. But it may cause several problems such as increase of resistance in the pathogen populations, toxicity to non-target organisms, environmental pollution, and the presence of residual chemicals in crops, which is harmful to the consumer health. For all these reasons, biological control via application of beneficial microorganisms can be effective in management of destructive phytopathogens (Taheri and Flaherty, 2022).

Fungal endophytes live in plant tissues for at least a part of their life cycle without causing disease symptoms (Dutta et al., 2014). These fungi have beneficial effects on the host plants, including plant growth promotion and induction of plant defense mechanisms which led to increased resistance against various biotic and abiotic stresses (Murthy et al., 2014; Daroodi et al., 2021a,b; Kheyri et al., 2022). The plant growth promoting fungi (PGPF) are capable of phosphate solubilization, production of indole-3-acetic acid (IAA), siderophore, and extracellular enzymes, which are involved in enhancing plant growth parameters (Jogaiah et al., 2013; Hossain et al., 2017; Zhang et al., 2018). Many studies have reported the ability of endophytic fungi to promote plant growth, which may be attributed to the production of secondary metabolites including phytohormones (as auxins and gibberellins) and siderophore, also the ability to solubilize nutrients for their host plants (Waqas et al., 2014; Khan et al., 2015; Numponsak et al., 2018).

The genus *Acrophialophora*, with *A. nainiana* as its type species, is considered as a thermotolerant and widely distributed fungal genus in temperate and tropical zones. This fungus is classified in Ascomycota and belongs to the *Chaetomiaceae* family. The genus *Acrophialophora* is previously reported as a biocontrol agent against several phytopathogens, such as *Pythium aphanidermatum* (Sharma et al., 1981; Ramzan et al., 2014), *Fusarium udum* (Rai and Upadhyay, 1983), *Phythium debaryanum*, *Phytophthora capsica*, *Sclerotinia sclerotiorum*, *Botrytis cinerea*, *Gaeomannomyces graminis*, *R. solani* (Turhan and Grossmann, 1989; Ramzan et al., 2014; Daroodi et al., 2021a), *Macrophomina phaseolina* (Siddiqui and Mahmood, 1992; Ramzan et al., 2014), *Fusarium solani* (Ramzan et al., 2014), and *Alternaria porri* (Abdel-Hafez et al., 2015).

Previous reports indicate the efficiency of fungal species as biocontrol agents against *Alternaria* spp. For example, *Penicillium oxalicum* was effective against *A. alternata* in rice (Sempere and Santamarina, 2010), *Trichoderma harzianum* was effective against *A. alternata* in quince (Tekiner et al., 2019), and also in strawberry and cucumber fruits (Tozlu et al., 2018). *Trichoderma viride* and *T. harzianum* were effective against *A. solani* in tomato (Roy et al., 2019). Also, the endophytic fungus *Chaetomium globosum* (Fayyadh and Yousif, 2019), and *Trichoderma asperelloides* (Ramírez-Cariño et al., 2020), *T. harzianum* and *T. longibrachiatum* (Hosseinmardi et al., 2020) were antagonistic against *A. alternata* in tomato.

Seed coating technology is one of the methods that increase germination rates, improve seed performance and protect the seeds and seedlings from pathogens. In this method, the seeds are treated using chemical, physical (such as hot water, dry or aerated heat and radiation) or biological agents, before planting. Seed treatments with biocontrol agents in different crops have been reported for control of diseases caused by *Pythium ultimum* (Callan et al., 1990; Mathre et al., 1995), *Penicillium oxalicum* (Mathre et al., 1995), *P. arrhenomanes*, and *Fusarium graminearum* (Mao et al., 1997) in corn, *Pythium* sp. on canola, safflower, dry pea, and sugar beet (Bardin et al., 2003), *Macrophomina phaseolina*, *Rhizoctonia solani* and *Fusarium* spp., on okra and sunflower plants (Dawar et al., 2008), *R. solani*, *Fusarium solani* and *Sclerotium rolfsii* in faba (El-Mougy and Abdel-Kader, 2008).

Also, plant growth promotion using endophytic fungi has been demonstrated in tomato plants by many researchers. For example, *Serendipita indica* (formerly *Piriformospora indica*) (Fakhro et al., 2010), *Trichoderma atroviride* and *Trichoderma hamatum* (Tucci et al., 2011), *Penicillium simplicissimum* (Khan et al., 2015), *Pochonia chlamydosporia* (Zavala-Gonzalez et al., 2015), *Neocosmospora haematococca* (formerly *Nectria haematococca*) (Valli and Muthukumar, 2018) and *Fusarium* spp. (Nefzi et al., 2019) have been reported as endophytic PGPF, which enhance growth parameters in tomato plants.

To our knowledge, plant growth promotion traits and biocontrol effects of *A. jodhpurensis* (*Chaetomium jodhpurense* Lodha) against *A. alternata* pathogenic on tomato plants have not been investigated, till now. Thus, the aims of this study were (i) to isolate and identify the endophytic fungus *A. jodhpurensis* (ii) to investigate effect of the endophyte on growth parameters of tomato plants and its association with phosphate solubilization, indole-acetic acid (IAA), siderophore, urease, and extracellular enzymes production (iii) to investigate effect of *A. jodhpurensis* on vegetative growth, spore germination, and hyphal structure of *A. alternata in vitro*, and (iv) to determine its effect on development of the disease caused by *A. alternata in vivo* using seed inoculation with spores of *A. jodhpurensis.*

## Materials and methods

### Endophytic fungus

#### Sample collection

Sampling was carried out in Mashhad, Razavi Khorasan province, Iran (36.2972°N, 59.6067°E) in September 2017. Briefly, 10 samples were randomly collected from healthy tomato plants by walking in a zig-zag pattern in one field. The samples were transported to the Plant Pathology Laboratory at Ferdowsi University of Mashhad in sterile plastic bags.

#### Isolation

The endophytic fungal isolate Msh5 was obtained from healthy roots of tomato in the north east of Iran using the method described by Wiyakrutta et al. (2004). Briefly, the roots were washed under running tap water, then cut into 1 cm fragments. These pieces were sterilized using 0.5% (w/v) sodium hypochlorite solution for 5 min, then 70% (v/v) ethanol for 1 min, and rinsed three times with sterile distilled water. The root pieces were dried on sterile filter paper and five pieces were placed in each petri dish containing potato dextrose agar (PDA) medium amended with streptomycin (250 mg/L) and chloramphenicol (250 mg/L) to inhibit bacterial growth. After incubation at 25°C for 12 days, the fungal isolate was purified on PDA using the hyphal tip method.

#### Morphological identification

The endophytic fungal isolate obtained in this study was cultured on three different media, including oat agar (OA), PDA, and 1/10-strength potato agar (1/10-strength PA, containing 20 g potato boiled for 30 min and filtrated, 15 g agar, and 1 l distilled water) and incubated in the dark at 25°C for 7 days. Morphological identification of the fungal isolate, Msh5, http://www.mycobank.org/BioloMICS.aspx?TableKey=14682616000000067&Rec=572180&Fields. All was performed on the basis of morphological characters of the colony, hyphae, and the characteristics of reproductive structures, according to the identification key described by Wang et al. (2019).

#### Molecular identification

The isolate Msh5 http://www.mycobank.org/BioloMICS.aspx?TableKey=14682616000000067&Rec=572180&Fields. All was cultured in potato dextrose broth (PDB) and incubated in a rotary shaker at 30°C and 150 rpm for 2 weeks. Then, the mycelia and spores of the fungus were harvested from the liquid culture using Whatman filter paper. Genomic DNA was extracted according to the method of Zhang et al. (2010) using cetyltrimethyl ammonium bromide (CTAB). Briefly, cell walls of fungal mycelia and spores were broken down by grinding via sterile mortar and pestle with liquid nitrogen until the dry powder was obtained. Then, CTAB extraction buffer (containing CTAB 10%, NaCl 5M, EDTA 20 mM, and Tris- HCL 1M) was added, and after incubation at 65°C for 45 min, purification was done with chloroform: isoamyl alcohol (24: 1). The mixture was centrifuged at 10,000 *g* for 15 min and the supernatant was taken, then isopropanol was added and was centrifuged at 13,000 *g* for 5 min. Finally, the DNA was rinsed with 70% (v/v) ethanol and dissolved in 50 μl of pure water.

The primers used for PCR amplification were ITS1 (TCC GTA GGT GAA CCT GCG G) and ITS4 (TCC TCC GCT TAT TGA TAT GC) (White et al., 1990), which amplified the ITS1-5.8S-ITS2 region of rDNA. Also, the primers T1 (AAC ATG CGT GAG ATT GTA AGT) (O’Donnell and Cigelnik, 1997) and TUB4Rd (CCA/G GAC/T TGA/G CCA/G AAA/G ACA/G AAG TTG TC) (Groenewald et al., 2013) were used for amplification of the partial beta-tubulin (*tub2*) gene region. Amplifications were performed in a total reaction volume of 25 μl, containing 13 μL master mix (parstous- Iran), 1 μL of each primer (10 pM), 8 μL deionized water, and 2 μL template DNA (50 ng). The PCR amplifications were performed in a thermal cycler (Biometra, Gottingen, Germany) with an initial denaturing step at 95°C (3 min), 35 cycles of denaturation at 95°C (30 s), annealing at 50°C (45 s), and extension at 72°C (90 s), followed by a final extension step at 72°C (10 min) for amplification of the ITS (Sekhar et al., 2018), and the *tub2* regions (Wang et al., 2016). The PCR products were investigated on 1% agarose gels and sequenced by Macrogen Co. (Seoul, Korea), using the ITS1 (for ITS1-5,8S-ITS2 region of rDNA) and TUB4Rd (for *tub2*) primers. Analysis of sequences was performed using the basic local alignment search tool (BLAST) program compared to the available data of the national center for biotechnology information (NCBI) Gene Bank databases to determine DNA similarities. The most similar sequences were downloaded and DNA sequences were manually edited using Bioedit v7.1.3 and aligned using ClustalW software. Phylogenetic analyses for the ITS and tub2 regions were done using the ITS1 and TUB4Rd sequences via the MEGA 7 software by maximum likelihood analysis methods based on the Tamura- Nei model and the percentages of the replicate branches with 1,000 bootstraps were shown next to the branches. The nucleotide sequences of the Msh5 isolate were submitted to the Gene Bank and the accession numbers were obtained for the ITS and beta-tubulin genomic regions.

### Fungal pathogen

The isolate of *Alternaria alternata*, which was previously isolated from infected tomato plants (Ramezani et al., 2019), was obtained from Department of Plant Protection, Faculty of Agriculture, Ferdowsi University of Mashhad in Iran.

### Plant growth promotion traits of the endophytic fungus *in vitro*

#### Indole-acetic acid production assay

Indole-acetic acid production by the endophytic fungus was evaluated via culturing a 9 mm diameter disk of *A. jodhpurensis* in basal salt medium (BSM) containing 5 g glucose, 1 g KH_2_PO_4_, 0.5 g MgSO_4_, 0.5 g KCl, 1 L distilled water, pH 5.5, amended with L-tryptophan (0.1, 0.01, 0.001, and 0 mg L^–1^) and was incubated on a rotary shaker at 30°C and 150 rpm for 7, 10, 15, and 20 days (Bose et al., 2013). Then, the medium was centrifuged (5000 rpm/min for 10 min), and the supernatant was mixed with Salkowski’s reagent (150 ml of concentrated H_2_SO_4_, 250 mL of distilled water, 7.5 ml of 0.5 M FeCl_3_⋅6H_2_O) with a 1:2 (v/v) ratio, and was kept at room temperature for 20 min. The pink color produced showed IAA production (Gordon and Weber, 1951), and the IAA concentration was measured at 530 nm using a spectrophotometer.

#### Phosphate solubilization assay

For phosphate solubilization assay, a 9 mm diameter disk of *A. jodhpurensis* was cultured on Pikovskaya’s medium (Pikovskaya, 1948), containing 0.5 g yeast extract, 10 g dextrose, 5 g Ca_3_(PO_4_)_2_, 0.5 g (NH_4_)_2_SO_4_, 0.1 g MgSO_4_, 0.02 g KCl, 0.02 g NaCl, 0.003 g MnSO_4_.H2O, 0.003 g FeSO_4_.7H2O, 15 g agar, in 1 L distilled water with pH 7.2. After incubation at 28°C for 5 days, a clear zone around the colony indicated phosphate solubilization.

Quantitative estimation of phosphate solubility was done using NBRIP (National Botanical Research Institute’s phosphate) medium containing 10 g glucose, 5 g Ca_3_(PO_4_)_2_, 5 g MgCl2 z 6H2O, 0.25 g MgSO_4_ 7 H2O, 0.2 g KCl, 0.1 g (NH_4_)_2_SO_4_ and 0.025 g bromophenol blue in 1 L distilled water (Mehta and Nautiyal, 2001). Un-inoculated medium was used as negative control. The flasks were incubated on a rotary shaker at 30°C and 150 rpm for 7, 10, 15, and 20 days. Then, the medium was centrifuged (5000 rpm/min for 10 min) and the supernatant was collected and autoclaved at 121°C for 20 min. Optical density was measured at 700 nm.

#### Production of urease and siderophore

Urease production assay was performed by culturing a 9 mm diameter disk of *A. jodhpurensis* on Christensen’s medium (containing 1 g peptone, 1 g glucose, 5 g NaCl, 2 g KH_2_PO_4_, 15 g agar, and 0.012 g phenol red in 1,000 mL distilled water, pH 6.8), which was sterilized for 10 min at 121°C. Then 0.5 mL of 20% seitz-filtered solution of urea was added to 4.5 mL sterile medium, when the medium cooled to 50°C (Seeliger, 1956). After incubation for 7 days at 28°C, the pink color produced indicated urease production.

The ability of *A. jodhpurensis* to produce siderophore was investigated using culturing a 9 mm diameter disk of *A. jodhpurensis* on the Chrome Azurol S (CAS)- agar medium (60.5 mg/50 mL Chrome Azurol S, 72.9 mg/40 mL HDTMA, which was mixed with 2.7 mg FeCl_3_,6H_2_O in 10 mM HCl, 42.23 g King’s B agar and 900 mL distilled water), as described by Milagres and Machuca (2003). Yellow to orange color indicated siderophore production.

#### Production of extracellular enzymes

The endophytic fungus *A. jodhpurensis* was grown on PDA at 28°C for 7 days. Then, a 9 mm diameter disk of this fungus was placed in Petri dishes containing specific medium for each enzyme. The plates were incubated at 28°C, then the zones of enzyme activity around the fungal colony were investigated.

For cellulase assay, the antagonistic fungus was grown on yeast extract peptone agar medium (0.1 g yeast extract, 0.5 g peptone, 16 g agar in 1 L distilled water) amended 0.5% Na-carboxymethyl cellulose (CMC) for 7 days at 28°C. Then, the plates were flooded with 0.2 aqueous Congo Red and destained with 1 M NaCl for 15 min (Lingappa and Lockwood, 1962). The clear zone around the fungal colony showed cellulase activity.

Lipase assay was performed by growing *A. jodhpurensis* for 7 days at 28 °C on peptone agar medium (10 g peptone, 5 g NaCl, 0.1 g CaCl2 2H2O, 16 g agar, 1 L distilled water; pH 6) amended with 1% tween 20. The clear zone around the fungal colony showed lipase production (Hankin and Anagnostakis, 1975).

For laccase assay, *A. jodhpurensis* was cultured on glucose yeast extract peptone (GYP) agar medium (1 g glucose, 0.1 g yeast extract, 0.5 g peptone, 16 g agar, 1 L distilled water, pH 6) supplemented with 0.005% 1- naphthol and incubated for 7 days at 28°C. Color of the medium changed from clear to blue around the fungal colony, which showed the laccase activity (Hankin and Anagnostakis, 1975).

For protease activity, *A. jodhpurensis* was grown on GYP agar medium supplemented with 0.4% gelatin (8 g gelatin in 100 ml distilled water, which was sterilized separately and mixed with sterile GYP agar medium, pH 6). After incubation for 7 days at 28°C, the plates were flooded with saturated aqueous ammonium sulfate. The clear zone around the colony indicated protease activity (Hankin and Anagnostakis, 1975).

For amylase activity, *A. jodhpurensis* was cultured on GYP agar medium supplemented with 2% soluble starch and incubated for 7 days at 28°C. Following treatment with 1% iodine in 2% potassium iodide, a clear zone around the colony showed amylase activity (Hankin and Anagnostakis, 1975).

### *In vitro* biocontrol activity of *Acrophialophora jodhpurensis*

#### Antagonistic activity assay

The isolate of *A. jodhpurensis* was tested for its antagonistic activity against *A. alternata* using the dual culture method on PDA medium. Briefly, *A. jodhpurensis* and *A. alternata* were grown separately on PDA plates at 28°C for 7 days. Then, a mycelial disk (9 mm diameter) of the *A. jodhpurensis* was cultured on one side of the petri dish containing PDA. A 9 mm disk of PDA (without any fungus) was used as control. After 2 days, a mycelial disk (9 mm diameter) of *A. alternata* was cultured on the other side of each Petri dish in the opposite side of the antagonist. The colony diameter of the pathogen was measured after 7 days and compared to the control (Sánchez-Fernández et al., 2016). The inhibitory effect of *A. jodhpurensis* on mycelial growth of the pathogen was calculated using the following formula (Asran-Amal et al., 2010):

L=((C-T)/C)×100

In this formula, L is the inhibition of radial mycelial growth, C and T are the colony diameters of *A. alternata* in the control and in presence of the antagonist, respectively.

#### Effect of volatile metabolites of the antagonistic fungus on *Alternaria alternata* growth

To investigate the antagonistic activity of volatile metabolites released from mycelia of *A. jodhpurensis* against *A. alternata*, the isolates of *A. jodhpurensis* and *A. alternata* were grown on PDA medium at 28°C for 7 days. Then, a mycelial plug (5 mm diameter) of *A. jodhpurensis* was placed in the center of the Petri dish containing PDA, and a mycelial plug (5 mm diameter) of *A. alternata* was cultured in the center of a second PDA medium. The Petri dish containing *A. jodhpurensis* was immediately inverted over the *A. alternata* Petri dish and the Petri dishes were rapidly sealed with parafilm and incubated at 28°C in the dark. The growth of *A. alternata* was measured and compared to the control after 7 days (Nishino et al., 2013).

#### Effect of non-volatile metabolites of the endophyte on the pathogen growth

Antifungal activity of non-volatile compounds of *A. jodhpurensis* was investigated as described by Xiao et al. (2013). Briefly, the Msh5 isolate of *A. jodhpurensis* was grown on PDA medium at 28°C for 7 days. Then, two mycelial plugs (10 mm × 10 mm) of this fungus were transferred into a flask containing 100 mL potato dextrose broth (PDB) medium. The flasks were incubated on a rotary shaker at 30°C and 150 rpm for 10 days. Control flasks containing 100 mL PDB were not inoculated with *A. jodhpurensis*. For preparing supernatant, the culture was filtered by Whatman filter paper (no. 1) for removing mycelia, then sterilized using a 0.2 μm pore biological membrane filter. The supernatant was added to PDA media at concentrations of 3, 6, 10, and 15% (v/v) (Li et al., 2015). Then, a 9 mm diameter mycelial plug from 7 days old culture of *A. alternata* was placed in the center of each PDA plate supplemented with the supernatant and maintained at 28°C. Colony diameter of the pathogen was determined after 7 days. The experiment was repeated three times with three replications for each repetition.

#### Effect of the antagonistic fungus on mycelial structure of *Alternaria alternata*

The hyphal structures of *A. alternata* in dual culture with *A. jodhpurensis* and in the PDA medium containing supernatant of the endophytic fungus in liquid culture were investigated using light microscopy. Briefly, a thin mycelial plug of *A. alternata* from the edge of colony was stained using aniline blue according to the method of Koneman et al. (1978) and checked by an Olympus microscope (BH2, Tokyo, Japan) when the control Petri dishes were completely covered with the pathogen mycelia.

#### Effect of *Acrophialophora jodhpurensis* on spore germination of the pathogen

The method described by Rex et al. (2019) was used for investigating effect of *A. jodhpurensis* on spore germination of *A. alternata.* Growth-free supernatant was prepared as mentioned above, and then it was used for investigating its effect on spore germination of the pathogen. The spore concentration of *A. alternata* was adjusted to 10^5^ spores mL^–1^ using a hemocytometer. Then, the supernatant (100, 70, 50, and 5% v:v, diluted with sterile distilled water) was mixed with spore suspensions of *A. alternata* at a ratio of 1:1 (v:v) (Li et al., 2015). The mixtures were incubated at 24°C with 90% relative humidity. After 24 h, germination of the spores was checked by an Olympus microscope (BH2, Tokyo, Japan). Spore germination was determined as the development of a germ tube to a length equal to one-half of the spore diameter (Medwid and Grant, 1984). The spore germination (spores/mL) was investigated as a percentage using the following formula (Li et al., 2015):

Totalgerminationrate(%)=(Germinatedspores÷Totalspores)×100

### *In vivo* assays

#### Inoculum preparation

The isolate of *A. alternata* was cultured on potato carrot agar (PCA) medium and incubated at 28°C for 7 days. Then, spore suspension of *A. alternata* was prepared by adding sterile distilled water into Petri dishes containing *A. alternata.* The spore concentration was adjusted to 10^6^ spore mL^–1^ using a hemocytometer. For inoculating tomato seedlings, the spore suspension was supplemented with 0.05% tween 20 (Ramezani et al., 2019).

For preparing *A. jodhpurensis* inoculum, this antagonistic fungus was grown on 1/10 strength PA (potato agar) and incubated at 28°C for 10 days (Su et al., 2012). The spore suspension of *A. jodhpurensis* was prepared by adding sterile distilled water into Petri dishes containing *A. jodhpurensis*, then ascospores were washed and spore concentration was adjusted to 10^7^ spore mL^–1^ using a hemocytometer (Daroodi et al., 2021a).

#### Seed inoculation with spores of *Acrophialophora jodhpurensis* and seed colonization test

Tomato seeds variety “Mobil” were surface-sterilized by 1% sodium hypochlorite for 2 min, then rinsed three times with sterile distilled water. Seed coating was done by spore suspension of *A. jodhpurensis* amended with 1% sugar, 0.5% carboxymethyl cellulose (CMC), or 0.5% molasses solution as sticker. Forty tomato seeds were transferred into each sterilized Petri dish containing 2 mL spore suspension, then dried in laminar flow. For control, the seeds were only treated with sterile distilled water containing 1% sugar, 0.5% carboxymethyl cellulose (CMC), or 0.5% molasses solutions.

After drying the seeds, 10 seeds treated with the spore suspension were placed in a test tube containing 9 mL sterilized distilled water for investigating seed colonization. The test tube was shaken and spore concentration was quantified by a hemocytometer.

#### Plant growth conditions

Tomato seeds variety “Mobil,” which were coated using spore suspension of *A. jodhpurensis* and stickers such as 1% sugar, 0.5% carboxymethyl cellulose (CMC), or 0.5% molasses solution, were planted in 12 × 10 cm pots containing sterilized perlite, soil and sand (1:2:1). One seed was planted in each pot and the pots were incubated under greenhouse conditions (30 ± 4°C with 16/8 h light/dark photoperiod).

#### Detecting *Acrophialophora jodhpurensis* in tomato roots and root colonization assay

Evaluation of tomato root colonization by *A. jodhpurensis* was done at 30 days post-inoculation (dpi). The plants were removed from the soil and washed using running tap water. Then, the roots were stained using cotton blue and investigated by an Olympus microscope (BH2, Tokyo, Japan) (Vierheilig et al., 1998).

To investigate tomato roots colonization by *A. jodhpurensis*, reisolation of the fungal endophyte from tomato roots was done. The roots were washed under running tap water for one min. Then, washed roots were surface-sterilized using 2% sodium hypochlorite for 2 min and then 70% ethanol for 2 min. One g of root pieces per plant were dissected 10 mm long. The root fragments were dried on sterile filter paper and four pieces were placed in each Petri dish (10 cm diameter) containing PDA medium amended with streptomycin (0.05%) and incubated at 25°C for 10 days. Root colonization was investigated by counting single colonies grown from root pieces using a dissecting microscope (Dingle and Mcgee, 2003).

#### Effect of *Acrophialophora jodhpurensis* on biocontrol of the disease caused by *Alternaria alternata*

After 30 days of planting tomato seeds (at four leaves growth stage), when the plant roots were colonized by *A. jodhpurensis* very well, effect of the antagonistic fungus on biocontrol of the disease caused by *A. alternata* was investigated *in vivo.* The plants were inoculated with *A. alternata* by spraying the plants using spore suspension of the pathogen at concentration of 10^6^ spore mL^–1^ containing 0.05% tween 20. For control, the tomato seedlings were only sprayed with sterile distilled water containing 0.05% tween 20. The inoculated seedlings were kept in the greenhouse at 90% relative humidity and 30 ± 4°C temperature. Three replications and three repetitions were used for each treatment.

Disease evaluation was performed at 7 days after *A. alternata* inoculation, when the plants were at 5–6 leaves growth stage.

Disease progress was graded into five classes on the basis of leaf spot development including 0 = no leaf spot symptoms; 1 = 1 –25% leaf area covered by disease symptoms; 2 = 26–50%; 3 = 51–75%; 4 = 76–99% and 5 = 100% leaf area covered by the symptoms (Kumar et al., 2011). The disease index was calculated as described by Taheri and Tarighi (2010). Each experiment was repeated three times with three replications for each repetition.

#### Effect of the endophytic fungus on plant growth promotion

To investigate the effect of *A. jodhpurensis* on tomato growth parameters, the seeds were inoculated with spore suspension of the endophyte and planted as described before. After 7 days, fresh and dry weight, shoot, and root length of plants were measured. To determine the dry weight, the samples were dried in an oven at 70°C. Three replications and three repetitions were maintained for each treatment.

### Statistical analysis

The assays were repeated at least three times with three replications in each repetition. The presented data for each assay were the means (±standard error) of three experiments. Data were analyzed using the Minitab 18 software using one-way analysis of variance (ANOVA). The means were separated by the Fisher test at the level of *P* ≤ 0.05. All diagrams were drawn using Excel 2013.

## Results

### Morphological and molecular identification of *Acrophialophora jodhpurensis*

**FIGURE 1:**
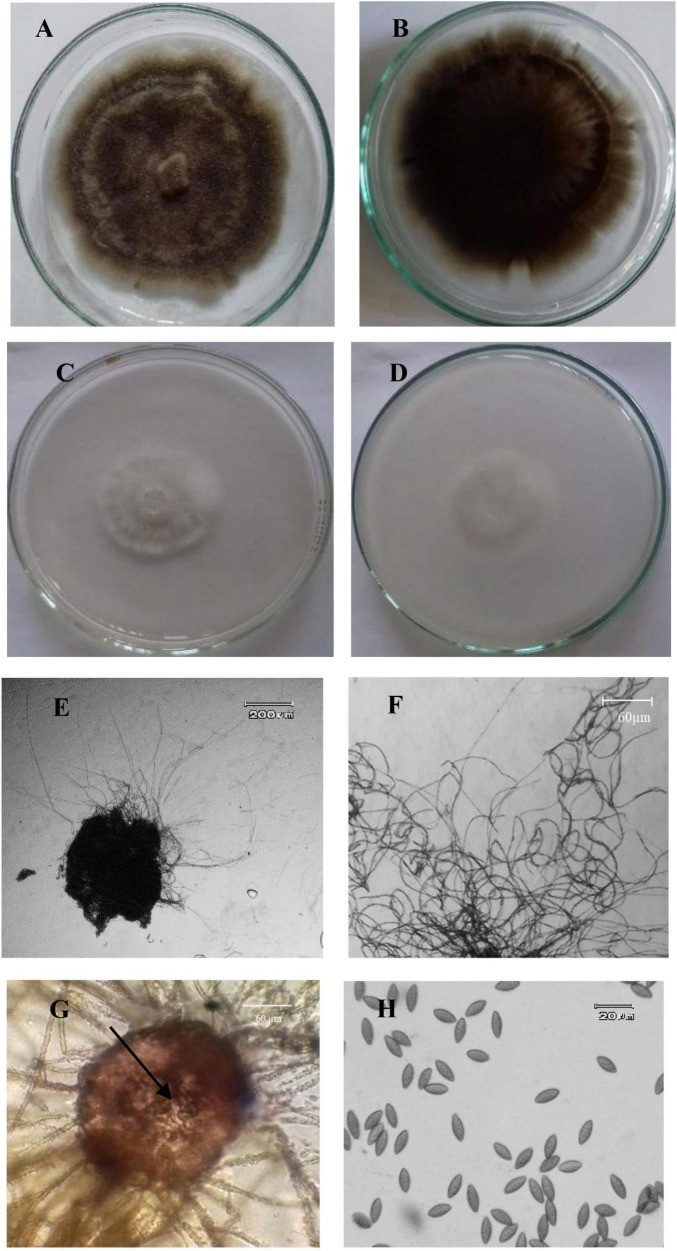
Morphological characteristics of *Acrophialophora jodhpurensis* (isolate Msh5). Colonies of *A. jodhpurensis* from up **(A)** and down **(B)** on potato dextrose agar (PDA), also up **(C)** and down **(D)** on oat agar (OA), ascomata of *A. jodhpurensis*, subglobosa or ovate, ostiolate **(E)**, flexuous or undulate hairs **(F)**, clavate, fasciculate or fusiform asci **(G)**, unicellular and fusiform ascospores of *A. jodhpurensis* **(H)**.

Among 15 endophytic fungi obtained from the sampled plants, six isolates belonged to *A. jodhpurensis.* The most antagonistic isolate of this endophyte (Msh5), which showed highest inhibitory effect against the pathogen compared to other endophytes obtained, was used for the rest of experiments. This isolate was identified based on morphological characteristics of the colony, hyphae, and reproductive structures including ascomata, ascomatal hairs, and ascospores (Wang et al., 2019). The colonies had 25 mm diameter on PDA (Figures 1A,B) and 25–31 mm diameter on OA after 7 days of growth at 25°C (Figures 1C,D). Ascomata were superficial, subglobosa to ovate, ostiolate, 130–220 × 100–180 μm and ascomatal wall was brown (Figure 1E). Ascomatal hairs were brown, flexuous, or undulate, rather long and thin, occasionally branched and 2–5 μm thick (Figure 1F). The asci were clavate, fasciculate or fusiform, with eight spores (Figure 1G). Ascospores were unicellular, not triangular in face view, often fusiform 12–16 × 6–8 μm and usually brown when mature (Figure 1H). Asexual stage was not observed.

**FIGURE 2:**
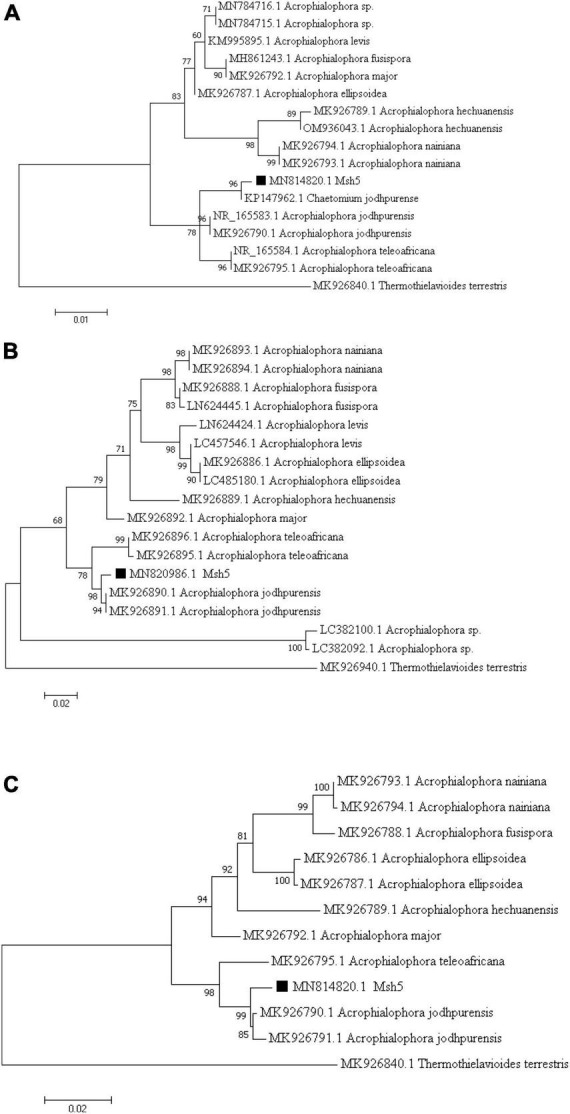
Phylogenetic tree based on the ITS region of rDNA and β-tubulin (*tub2*) sequences of the endophytic fungal isolate Msh5 obtained from tomato roots in Khorasan Razavi province of Iran. The trees are drawn using the maximum likelihood method based on the Tamura- Nei model in MEGA7 with 1,000 Bootstrap, in which the percentage of replicate trees is shown above the branches, based on the ITS **(A)**, β-tubulin **(B)** sequences and phylogenetic tree based on the combined ITS and β-tubulin **(C)**, which showed the relationship between the Msh5 isolate and other fungi. The trees are rooted with *Thielavia terrestris* (syn *Thermothielavioides terrestris*) as the out-group, which is classified in the Ascomycota and belongs to Sordariales.

Based on molecular analysis of the internal transcribed spacer (ITS) and β-tubulin (*tub2*) genomic regions, the Msh5 isolate was identified as *A. jodhpurensis* (*C. jodhpurense* Lodha). The phylogenetic trees were obtained using the neighbor-joining method with 1,000 bootstraps and the percentage of replicate trees were shown above the branches (Figures 2A–C). Accession numbers for the ITS and *tub2* sequences, including MN814820 and MN820986 respectively, were obtained from National Center for Biotechnology Information (NCBI).

### Production of indole-acetic acid

**FIGURE 3:**
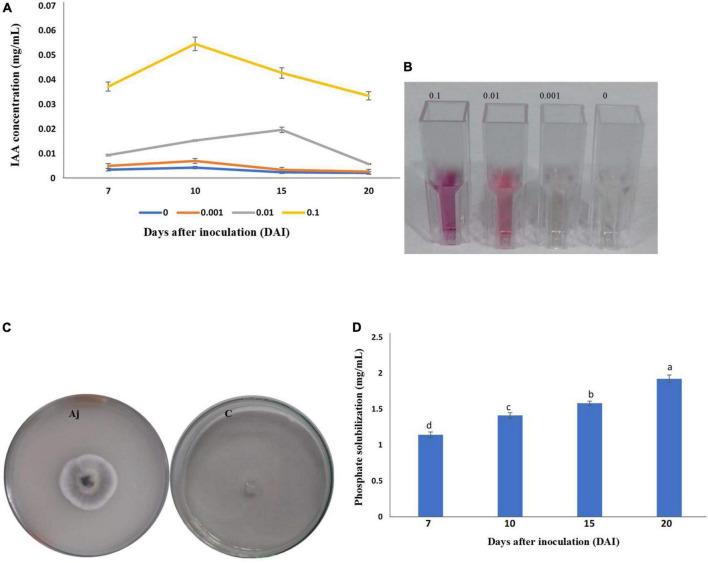
Production of indole-3-acetic acid (IAA) and phosphate solubilization by the isolate Msh5 of *Acrophialophora jodhpurensis.* Production of IAA by *A. jodhpurensis* in basal salt medium (BSM) amended with 0.1, 0.01, 0.001, and 0 mg L^–1^ L-tryptophan at various time points after inoculation **(A)**, production of pink color (IAA production) at 10 days after inoculation **(B)**, the clear zone around the *A. jodhpurensis* colony on Pikovskaya’s medium **(C)**, and the amount of phosphate released in NBRIP medium containing 0.025 g bromophenol blue in 1 L at various time points after inoculation of *A. jodhpurensis* **(D)**.

The isolate of *A. jodhpurensis* had the ability to produce IAA, as development of pink color showed IAA production. For investigating the effect of L-tryptophan on IAA production at various time points (7, 10, 15, and 20 days) after inoculation of *A. jodhpurensis*, different concentrations of L- tryptophan (0, 0.001, 0.001, and 0.1 mg L^–1^) were supplemented in the medium. Quantification of IAA was performed by measuring the absorbance at 530 nm using spectrophotometer. The spectrophotometric analysis revealed that the IAA production was related positively with L-tryptophan concentration. The maximum IAA production by *A. jodhpurensis* was observed at 10 days after inoculation when the medium was amended with 0.1 mg L^–1^ L-tryptophan (Figures 3A,B).

### Phosphate solubilization

Qualitative estimation of phosphate solubility was studied on Pikovskaya’s medium. The clear zone around the *A. jodhpurensis* colony showed phosphate solubilization capability of this fungus (Figure 3C). Also, quantitative estimation of phosphate solubilization was done using NBRIP medium containing 0.025 g bromophenol blue in 1 L distilled water at various time points (7, 10, 15, and 20 days) after inoculation of *A. jodhpurensis*. The spectrophotometric analysis showed that the maximum phosphate solution at 20 days after inoculation (Figure 3D).

### Production of urease and siderophore

**FIGURE 4:**
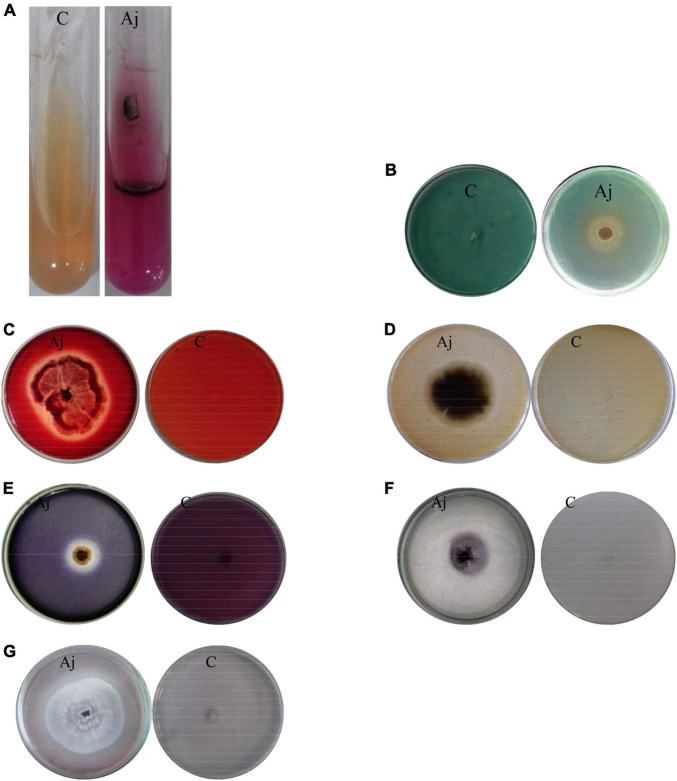
Investigating biocontrol traits of *Acrophialophora jodhpurensis* (isolate Msh5). Production of urease **(A)**, siderophore **(B)**, cellulase **(C)**, protease **(D)**, amylase **(E)**, laccase **(F)**, lipase **(G)**. C, negative control; Aj, *A. jodhpurensis.*

The ability of production of urease and siderophore was investigated on Christensen’s medium and Chrome Azurol S (CAS)-agar medium, respectively. The isolate of *A. jodhpurensis* had the ability to produce urease and siderophore. Development of pink color showed urease production (Figure 4A), and yellow to orange color indicated siderophore production (Figure 4B).

### Production of extracellular enzymes

The obtained data showed that the Msh5 isolate of *A. jodhpurensis* produced cellulase (Figure 4C), protease (Figure 4D), amylase (Figure 4E), laccase (Figure 4F), and lipase (Figure 4G) enzymes.

### Antagonistic activity of *Acrophialophora jodhpurensis* against *Alternaria alternata* in dual culture

**FIGURE 5:**
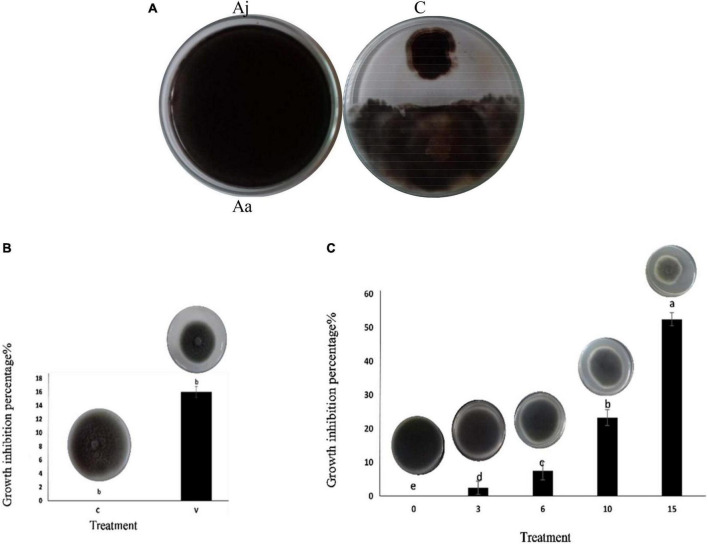
Inhibitory effect of *Acrophialophora jodhpurensis* (isolate Msh5) on mycelial growth of *Alternaria. alternata*. Biocontrol effect in dual culture test on potato dextrose agar medium **(A)**, the effect of volatile metabolites **(B)**, and growth-free supernatant (non-volatile metabolites) at 0, 3, 6, 10, and 15% (v/v) concentrations **(C)** after 7 days. Aa, *A. Alternata;* Aj, *A. jodhpurensis*; C, control; V, volatile metabolites.

The Msh5 isolate of *A. jodhpurensis* inhibited *in vitro* growth of *A. alternata* in dual culture on PDA. The inhibitory percentage (IP) of *A. alternata* growth was 50% using this beneficial endophytic fungus compared to the control (Figure 5A).

### Antifungal effect of volatile and non-volatile metabolites against *Alternaria alternata*

The obtained results revealed that *A. jodhpurensis* produced volatile and non-volatile metabolites, which inhibited *A. alternata* growth. The inhibitory percentage (IP) of *A. alternata* growth by volatile metabolites of *A. jodhpurensis* was 12.66 (Figure 5B). Also, the effects of growth-free supernatant of *A. jodhpurensis* at 3, 6, 10, and 15% (V: V) concentrations were investigated on *A. alternata* growth, which the IPs were 2.5, 7.5, 23.33, and 52.5 respectively. The 15% concentration was superior to the other concentrations tested in stopping the mycelial growth of *A. alternata* (Figure 5C).

### Microscopic observation of *Alternaria alternata* hyphae affected by *Acrophialophora jodhpurensis*

**FIGURE 6:**
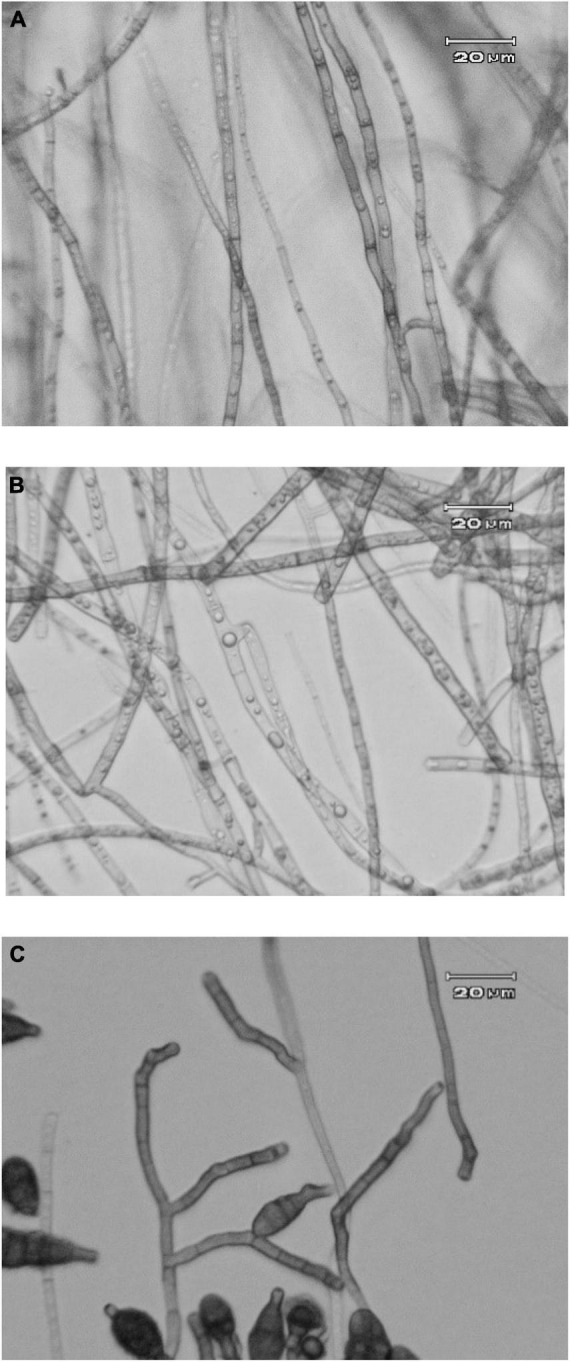
Optical microscope images of *Alternaria alternata* in dual culture with *Acrophialophora jodhpurensis* (isolate Msh5) on potato dextrose agar (PDA) medium containing supernatant (metabolites) of *A. jodhpurensis* when the control Petri dishes completely covered with the pathogen mycelia. The mycelia formation of *A. alternata* in dual culture with *A. jodhpurensis* **(A)** and on PDA containing supernatant at of *A. jodhpurensis* at 15% concentration **(B)**. The hyphae of *A. alternata* in control **(C)**.

Light microscopic analyses of the pathogen structures were done in dual culture with the antagonistic fungus and PDA medium containing growth-free supernatant at 15% concentration when the control Petri dishes were completely covered with the pathogen mycelia (after 7 days). Observations showed that the mycelium formation of *A. alternata* changed in presence of the antagonist and/or its metabolites. Production of colorless hyphae, deformation of hyphae and cytoplasm lysis was observed in the mycelia of *A. alternata* treated with the antagonistic fungus (Figure 6A), or its metabolites (Figure 6B) compared to the controls (Figure 6C).

### Effect of metabolites produced by endophyte on *Alternaria alternata* spore germination

**FIGURE 7:**
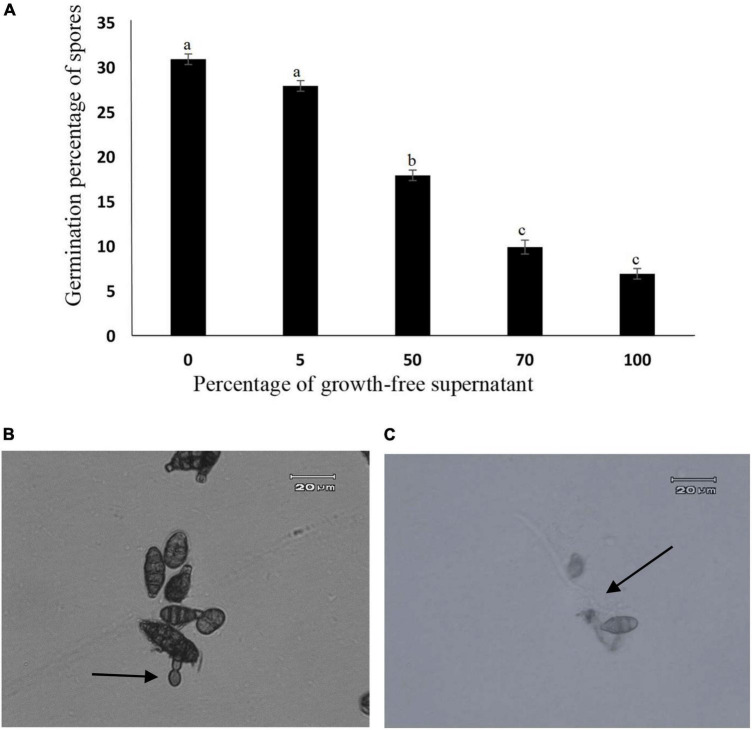
Effect of *Acrophialophora jodhpurensis* (isolate Msh5) supernatant on spore germination of *Alternaria alternata* after 24 h. Effect of *A. jodhpurensis* metabolites on germination percentage of *A. alternata* spores **(A)**, germ tube vacuolization and abnormal germination **(B)**, and normal germ tube formation in control **(C)**. Error bars correspond to standard error (SE) of three experiments.

The growth-free supernatant of *A. jodhpurensis* reduced spore germination of *A. alternata*. The concentration of 100% was superior to the other concentrations tested in reducing the spore germination of *A. alternata*, which reduced spore germination to 7% compared to the control which was 31% (Figure 7A). Also, spores of *A. alternata* produced germ tubes showing vacuolization and abnormal structure in presence of the supernatant (metabolites) of *A. jodhpurensis* (Figure 7B), compared to the controls (Figure 7C).

### Investigating colonization of tomato roots by *Acrophialophora jodhpurensis*

**FIGURE 8:**
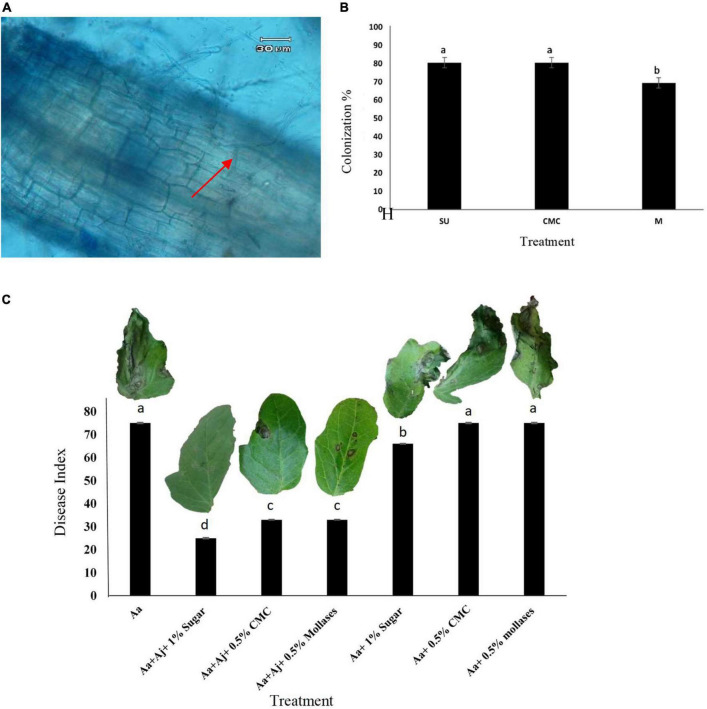
Detection of *Acrophialophora jodhpurensis* (isolate Msh5) in the colonized tomato roots at 30 days post inoculation **(A)**, colonization of tomato roots with spore suspension of *A. jodhpurensis* containing 1% sugar (SU), 0.5% carboxymethyl cellulose (CMC) and 0.5% molasses (M) solution as sticker at 30 days post inoculation **(B)** and effect of tomato seed coating with spore suspension of *A. jodhpurensis* and stickers on progress of the disease caused by *Alternaria alternata* on tomato seedlings **(C)**. Aa, inoculation with *A. alternata;* Aj, inoculation with *A. jodhpurensis;* H, *A. jodhpurensis* hyphae.

Intracellular hyphae of *A. jodhpurensis* were observed in microscopic analyses of tomato roots at 30 days post-inoculation (dpi) (Figure 8A). Colonization percentage of tomato roots by the endophytic fungal isolate was determined using seed coating with spores of *A. jodhpurensis* and 1% sugar (SU), 0.5% Carboxymethyl cellulose (CMC) and 0.5% molasses (M) solution as sticker. The data indicated that in seed coating with spores of *A. jodhpurensis*, sugar and carboxymethyl cellulose (CMC) were found to be the most effective for colonization of tomato roots, compared to molasses as sticker (Figure 8B).

### Effect of seed coating on biocontrol of *Alternaria alternata in vivo*

Tomato seed coating with *A. jodhpurensis* showed efficiency of this fungal endophyte in control of the disease caused by *A. alternata* on the seedlings. Among different coating materials used as stickers, sugar was found to be the most effective in controlling the pathogen compared to the controls and the plants only inoculated with *A. alternata* (Figure 8C).

### Effect of *Acrophialophora jodhpurensis* on plant growth promotion *in vivo*

Seed coating with spores of *A. jodhpurensis* together with stickers significantly increased growth characteristics of tomato seedlings, such as shoot fresh and dry weights, root fresh and dry weights, shoot and root lengths. The biomass enhancement was obvious in the plants inoculated with *A. jodhpurensis*, but the plants inoculated with *A. jodhpurensis* and 1% sugar showed more promising results in increasing shoot length and weight, and also root length and weight compared to the controls (Table 1).

**TABLE 1:** Effect of tomato seed coating with spores of *Acrophialophora jodhpurensis* (isolate Msh5) together with 1% sugar, 0.5% Carboxymethyl cellulose (CMC), and 0.5% molasses solutions as sticker on plant growth promotion.

Treatments	Shoot fresh weight (mg)	Root fresh weight (mg)	Shoot dry weight (mg)	Root dry weight (mg)	Shoot height (cm)	Root height (cm)
Water	592.3 ± 43.53 c	95.667 ± 5 e	51.33 ± 3.59 c	9.667 ± 0.89 c	8.400 ± 0.31 c	9 ± 0.5 bc
Aj + 1% Sugar	1230.3 ± 73.7 a	176.00 ± 5.97 a	122.33 ± 8.02 a	20.33 ± 1.22 a	11.300 ± 0.56 a	11.333 ± 0.89 a
Aj + 0.5% CMC	1038.7 ± 31.29 b	160.667 ± 5.30 b	100.33 ± 4.33 b	17.667 ± 0.67 b	11.167 ± 0.44 ab	10 ± 0.29 b
Aj + 0.5% Mollases	1004.7 ± 27.11b	122.33 ± 9.04 c	98.67 ± 3.24 b	17 ± 0.58 b	10.167 ± 0.44 b	9.5 ± 0.16 bc
1% Sugar	617 ± 33.35 c	106.00 ± 7.15 d	54.33 ± 3.44 c	10.333 ± 0.33 c	8.475 ± 0.23 c	8.967 ± 0.55 bc
0.5% CMC	557.3 ± 15.64 c	101.00 ± 5.29 de	59.67 ± 1.48 c	9 ± 1.018 c	8.667 ± 0.33 c	8.667 ± 0.17 c
0.5% Mollases	591.3 ± 74.94 c	96.33 ± 2.37 e	50 ± 2.94 c	8.667 ± 0.89 c	8.500 ± 0.29 c	8.833 ± 0.44 bc

The presented data for each assay are the means (±standard error) of three experiments. Statistical analysis was performed with Minitab 18 software. Aj, inoculation with A. jodhpurensis. Different letters indicate statistically significant differences according to Fisher analysis.

## Discussion

In this study, effect of the Msh5 isolate of *A. jodhpurensis* was investigated on plant growth promotion and biological control of *A. alternata* pathogenic on tomato both *in vitro* and *in vivo* conditions. The obtained data indicated that *A. jodhpurensis* produced IAA, siderophore and urease, also this fungus had the ability of solubilizing phosphate. Maximum production of IAA was observed at 10 days after *A. jodhpurensis* inoculation by adding L-tryptophan at 0.1 mg L^–1^ concentrations to the medium. Similar to our results, Bose et al. (2013) studied production of IAA by the white-rot fungus *Pleurotus ostreatus* using different concentrations of L-tryptophan in BSM, which the maximum production of IAA was observed by addition of L-tryptophan at 0.1 mg L^–1^ concentration.

The data showed that *A. jodhpurensis* produced a weak halo zone in the Pikovskaya’s medium, while this fungus in the liquid culture experiment (NBRIP medium containing bromophenol blue) had considerable phosphate solubilization capability. Similarly, Li et al. (2019) reported that only 30 of the 43 inorganic phosphate-solubilizing bacterial strains exhibited clear halo zones in the Pikovskaya’s medium, but all strains were able to dissolve tricalcium phosphate in the liquid culture.

Dual culture assay revealed the antagonistic capability of *A. jodhpurensis* against *A. alternata*. To our knowledge, this is the first report on the antagonistic effects of *A. jodhpurensis* against *A. alternata in vitro.* Many researchers studied antagonistic activities of *Acrophialophora* species against several phytopathogens *in vitro*. For example, *A. fusispora* was effective against *F. udum* (Rai and Upadhyay, 1983), *M. phaseolina* (Siddiqui and Mahmood, 1992), *F. solani*, *M. phaseolina*, *P. aphanidermatum*, *R. solani* and *S. rolfsii*, and *A. porri* (Demirci et al., 2011; Abdel-Hafez et al., 2015). *Acrophialophora levis* was antagonistic against *P. debaryanum*, *P. capsica*, *S. sclerotiorum*, *B. cinerea*, *Gaeomannomyces graminis*, and *R. solani* (Turhan and Grossmann, 1989), and *A. jodhpurensis* had antagonistic effect against *R. solani* (Daroodi et al., 2021a).

Beneficial fungi protect plants against pathogens via several mechanisms, such as mycoparasitism, antibiosis, competition, and induction of resistance (Zhang et al., 2012; Ghorbanpour et al., 2018). Our previous studies showed that *A. jodhpurensis* decreased the disease severity of *R. solani* not only directly by interacting with the pathogen, but also indirectly by inducing resistance mechanisms in tomato seedlings. Also, the Msh5 isolate of *A. jodhpurensis* induced systemic resistance in tomato against *A. alternata* via increasing phenolic contents, lignin accumulation, cell membrane stability, relative water content, accumulations of hydrogen peroxide, superoxide and iron ions, together with induction of antioxidant enzymes (such as catalase, guaiacol peroxidase, ascorbate peroxidase and superoxide dismutase) (Daroodi et al., 2021a,b).

In this study, *A. jodhpurensis* produced volatile and non-volatile metabolites, which inhibited *A. alternata* growth. Similar to these findings, production of volatile organic compounds (VOCs) with antifungal effects by the fungi belonging to the Chaetomiaceae family has been reported by several researchers (Bjurman and Kristensson, 1992; Korpi et al., 1998; Wady and Larsson, 2005). The VOCs, such as geosmin, 2-phenylethanol and phenylacetaldehyde were detected using gas chromatography- mass spectrometry (GC-MS) (Kikuchi et al., 1981). Also, volatiles of *Chaetomium thermophile* inhibited conidial germination and mycelial growth of *Humicola lanuginosa* (Satyanarayana and Johri, 1981) and the VOCs produced by *A. jodhpurensis* reduced mycelial growth of *R. solani* AG 4-HG II (Daroodi et al., 2021a).

In this study, the obtained data indicated that the growth-free supernatant (non- volatile metabolites) of *A. jodhpurensis* reduced mycelial growth of *A. alternata.* Similar to our results, immersing cucurbit fruits in growth-free supernatant of *A. nainiana* before inoculation of *Pythium* spp. delayed the disease symptoms (Sharma et al., 1981) and metabolites of *A. jodhpurensis* decreased mycelial the mycelial growth of *R. solani* AG 4-HG II (Daroodi et al., 2021a).

Also, the effects of *A. jodhpurensis* on spore germination and hyphal structure of *A. alternata* were studied using light microscopy. The data revealed that growth-free supernatant of *A. jodhpurensis* reduced spore germination of *A. alternata.* Deformation of hyphae and cytoplasm lysis were observed in the pathogen structures grown in the presence of *A. jodhpurensis* or its metabolites. Similar to these results, our previous study revealed that *A. jodhpurensis* decreased the growth and sclerotia production of *R. solani.* Also, growth patterns of *R. solani* in presence of the metabolites produced by *A. jodhpurensis* were different compared to the controls (Daroodi et al., 2021a).

To our knowledge, there is no information about effect of the other *Acrophialophora* species on spore germination and hyphal structures of pathogens. But in the Chaetomaceae family, in the interaction between *R. solani* and *Chaetomium spirale*, overgrowth of *C. spirale* on the colony of *R. solani*, coiling of *C. spirale* around *R. solani* hyphae and intracellular growth of the antagonist in the mycelia of *R. solani* occurred frequently. Also, in the advanced stage of interaction between the antagonist and the pathogen, the growth and development of *C. spirale* were associated with morphological changes of the pathogen, such as retraction of the plasma membrane and cytoplasm disorganization (Gao et al., 2005).

Effects of *A. jodhpurensis* on development of the disease symptoms caused by *A. alternata* were studied via seed coating using spore suspension of the biocontrol agent. The obtained results revealed a significant decrease in the disease index on tomato seedlings treated with *A. jodhpurensis* compared to the controls (non-treated with *A. jodhpurensis*). Similar to our results, seed coating using antagonistic fungi reduced the disease severity of several pathogens in various plans. For example, seed coating by *C. globosum* reduced *Fusarium roseum* f. sp. *cerealis* infection in corn (Chang and Kommedahl, 1968; Kommedahl and Mew, 1975), *Fusarium solani* f. sp. *pisi* in squash, snap bean, and pea (Hubbard et al., 2011), and seed coating with *C. globosum* and *C. funicola* reduced the infection of *Erysiphe graminis* f. sp. *hordei* in barley (Vilich et al., 1998). Also, seed coating with *C. globosum, C. cochiodes*, and *Chaetomium* sp. was effective against *Helminthosporium victoriae* in oat (Tveit and Moore, 1954), and treating tomato seeds with *Trichoderma harzianum* and *T. hamatum* was effective against *Fusarium* sp., *Verticillium* sp. and *Alternaria* spp. (El-Rafai et al., 2003). In addition, seed coating with endophytic fungus *Pestalotiopsis microspore* induced plant defense responses against *A. solani* in tomato (Sujatha et al., 2021).

Also, the effects of *A. jodhpurensis* on growth parameters of tomato seedlings were investigated using seed coating method. The obtained results revealed a significant increase in growth parameters of tomato seedlings treated with *A. jodhpurensis* compared to the controls (un-inoculated with *A. jodhpurensis*(. Similarly, inoculation of tomato roots by *Piriformospora indica* increased tomato fruit biomass in hydroponic culture (Fakhro et al., 2010). Inoculation of tomato roots using three grams of the *Nectria haematococca* inoculum containing 6 × 10^6^ colony forming units per gram significantly improved all the plant growth parameters including biomass of drought-stressed tomato, leaf characteristics and the plant height. Also, proline accumulation in shoots of the treated plants was significantly higher than untreated plants (Valli and Muthukumar, 2018). Inoculation of tomato seedlings using dipping in conidial suspension of *Withania somnifera* enhanced the plant growth parameters (Nefzi et al., 2019). Also, applications of *Ampelomyces* sp. and *Penicillium* sp. endophytes showed efficiency in enhancing plant growth, stress tolerance, recovery, and fruit yield (Morsy et al., 2020).

Also, many studies showed that treatment of tomato seeds with endophytic fungi improved plant growth parameters. Combined inoculation of *Glomus intraradices* and *Trichoderma atroviride* synergistically increased biomass production (Colla et al., 2015).

The results showed that seed coating with spores suspension of *A. jodhpurensis* containing sugar and CMC can be the most effective for colonization of tomato roots. Sugar supplement with *A. jodhpurensis* was found to be the most effective in the control of pathogen and also in plant growth promotion. These data may be associated with the role of sugar signaling in plant physiology and defense responses. Similarly, Morkunas and Ratajczak (2014) reported the importance of sugars in plant resistance.

## Conclusion

This study demonstrated considerable antagonistic activity of *A. jodhpurensis* against *A. alternata*, the causal agent of tomato early blight, *in vitro* and *in vivo* conditions. Also, the endophytic fungus *A. jodhpurensis* increased growth parameters of tomato plants. Therefore, this beneficial fungus could be used as a biological fertilizer and biocontrol agent for plant growth promotion and protection against fungal pathogens. Future studies seem to be necessary to investigate effects of *A. jodhpurensis* against other Alternaria species and various phytopathogens, human and animal safety assays, and finally formulation of this beneficial fungus or its metabolites and application in the field conditions.

## Data availability statement

The sequencing data presented in this study are deposited in NCBI with the accession numbers: MN814820 and MN820986.

## Author contributions

ZD and PT: study conception and design and manuscript preparation. ZD: performing the experiments and data collection. PT and ST: data analysis and interpretation of results. All authors reviewed the results and approved the final version of the manuscript.
